# Radiological spectrum of invasive mucormycosis in COVID-19

**DOI:** 10.1259/bjrcr.20210111

**Published:** 2022-03-09

**Authors:** Nandini Passi, Anshu C Wadhwa, Swati Naik

**Affiliations:** 1 Department of Radio-diagnosis, Batra Hospital and Medical Research Centre (BHMRC), New Delhi, India

## Abstract

Mucormycosis, commonly known as the “black fungus” is recently emerging as a deadly complication in COVID patients in the Indian subcontinent. A growing number of cases are being reported from all over the country, with a majority of the patients either undergoing treatment or having recovered from COVID.

Here, we report three cases of multisystem mucormycosis in COVID positive patients showing, rhino-orbital, cerebral, pulmonary, and genitourinary involvement. The first is a case of a 41-year-old male patient who during his treatment developed left periorbital swelling with ecchymosis and headache. CT and CE-MRI of the paranasal sinuses and brain revealed features of pan fungal sinusitis and subsequent invasion into the left orbit. The second case is of a 52-year-old male patient who after complaining of a severe left-sided hemicranial headache was diagnosed with cavernous sinus thrombosis. The third is of a 57-year-old male patient who presented with left flank pain and dysuria. HRCT (High-resolution CT) chest revealed a thick-walled cavitary lesion, and NCCT KUB (Non-contrast CT of Kidneys, ureters, and bladder) revealed left-sided pyelonephritis. A cystoscopic and microbiological evaluation revealed fungal growth.

In all three patients, a biopsy from the involved area revealed broad aseptate filamentous fungal hyphae suggestive of mucormycosis, which was confirmed on culture.

These are all unusual cases and physicians should be aware of the possibility of secondary invasive fungal infections in patients with COVID-19 infection.

## Case 1

A 41-year-old male patient, with newly detected diabetes mellitus, was admitted with a 5-day history of fever, dry cough, and weakness. He was febrile (103°) on admission, respiratory rate was 35/min, oxygen saturation of 92% on room air. He was initiated on 3–4 L/min of oxygen. The relevant physical examination revealed bilateral crepts at the lung bases with a normal cardiovascular and neurological exam.

A reverse transcriptase polymerase chain reaction (RT-PCR) from a nasopharyngeal and oral swab was positive for the SARS-CoV-2 virus.

His lab parameters on Day 1 of admission showed CRP- 24 mg l^−1^, ferritin >2000 ng ml^−1^, and HbA1c 10.1% signifying uncontrolled diabetes.

**Figure 1.**
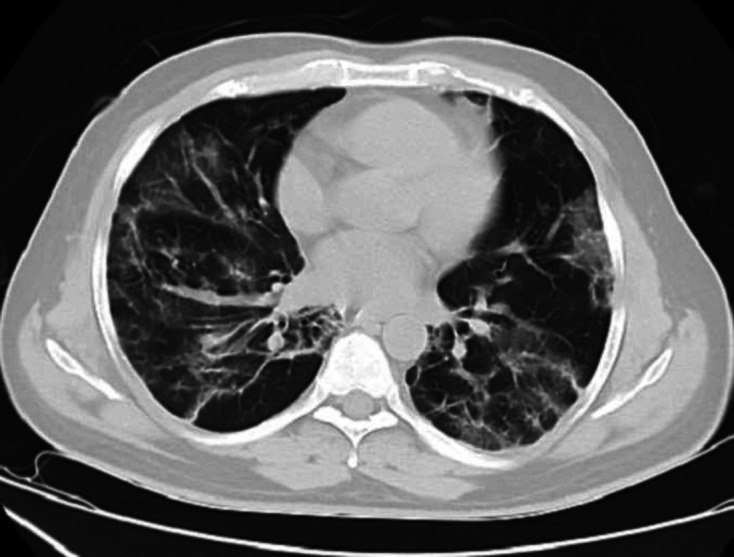
HRCT CHEST (axial section, lung window)- multiple ground-glass opacities, with a subpleural predominance- suggestive of COVID pneumonia.HC,

A CT scan of the chest on Day 2 of admission (Day 7 from initiation of symptoms) showed multifocal ground-glass opacities in both lungs predominantly in a peripheral distribution, strongly suggestive of COVID pneumonitis, with a CT severity score: 12/25. (Figure 1)

On Day 6, the patient showed worsening of symptoms evidenced as dyspnoea, tachypnoea, and a fall in oxygen saturation. His X-ray chest showed an increase in the inhomogenous opacities in bilateral lung fields, signifying worsening pneumonia, following which he was put on 15 L/min of supplemental oxygen.

He was then initiated on parenteral methylprednisolone 80 mg/day (0.5–1 mg/kg body weight)

**Figure 2.**
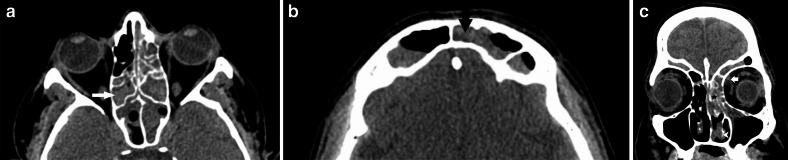
a, b: NCCT PNS (axial sections) showing mucosal thickening in bilateral ethmoid, sphenoid and frontal sinuses with hyperdense contents (arrows) - suggestive of fungal sinusitis. c: NCCT PNS (coronal section) depicting left inferior turbinate hypertrophy (arrowhead), mucosal thickening with hyperdense contents in the left ethmoid air cells (star). A small left-sided intraorbital soft tissue opacity (white arrow) in the extraconal compartment, reaching the medial rectus muscle. NCCT, non-contrast CT; PNS, paranasal sinus.

2 days after initiation of steroids (Day 9 of admission), he complained of nasal congestion and blockade, left eye swelling with pain and ecchymosis, and severe headache, not subsiding with non-steroidal anti-inflammatory drugs. However, the vision and ocular movements were maintained. A non-contrast CT PNS (paranasal sinuses) (Figure 2) was done the following day (Day 10), which showed mucosal thickening with hyperdense foci in the left nasal cavity, bilateral maxillary, ethmoid, sphenoid and frontal sinuses, raising suspicion for fungal sinusitis. An intraorbital soft tissue density was also noted in the left orbit, in the extraconal compartment, abutting the medial rectus muscle. However, there was no evidence of any obvious osseous erosion. The orbital apex and the pterygopalatine fossa appeared free of the disease.

**Figure 3.**
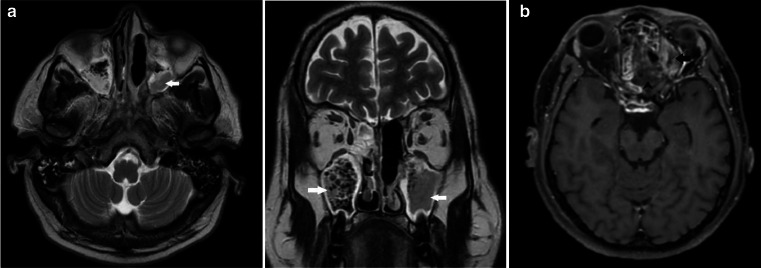
a- Axial and coronal, *T*_2_ weighted MRI - hypointense contents with signal voids are noted within bilateral maxillary (arrows) and ethmoid sinuses - suggestive of fungal sinusitis. b Post-contrast *T*_1_ weighted (axial section) MRI - Non-enhancing necrosed mucosa in the ethmoid air cells on the left side (arrowhead) with intraorbital extension (arrow).

The following day (Day 11), a contrast-enhanced MRI of Brain and PNS was done (Figure 3). Post-contrast images revealed diffuse nodular mucosal enhancement within all the sinuses, with T2 hypointense areas. Non-enhancing (necrosed) mucosa was seen within the left ethmoid air cells (very characteristic of invasive fungal sinusitis).

The intraorbital soft tissue seen on CT was better depicted on MRI. It was seen reaching up to the medial rectus muscle in the extraconal compartment, and obliterating the fat planes with it. There was significant increase in the orbital soft tissue in comparison to the CT done a day prior. The optic nerve and the orbital apex appeared normal.

The left cavernous sinus appeared mildly bulky, however, no obvious filling defect was noted and the normal flow voids were maintained.

Keeping in mind the current spike in the cases of mucormycosis (MCR) in the country, patient was taken up for diagnostic FESS (functional endoscopic sinus surgery) for a biopsy and surgical debridement was performed.

Microbiological analysis of the tissue sample revealed broad aseptate filamentous fungal hyphae, with characteristic growth patterns on culture, suggestive of MCR.

Patient was initiated on liposomal Amphotericin B (5 mg/kg)

**Figure 4.**
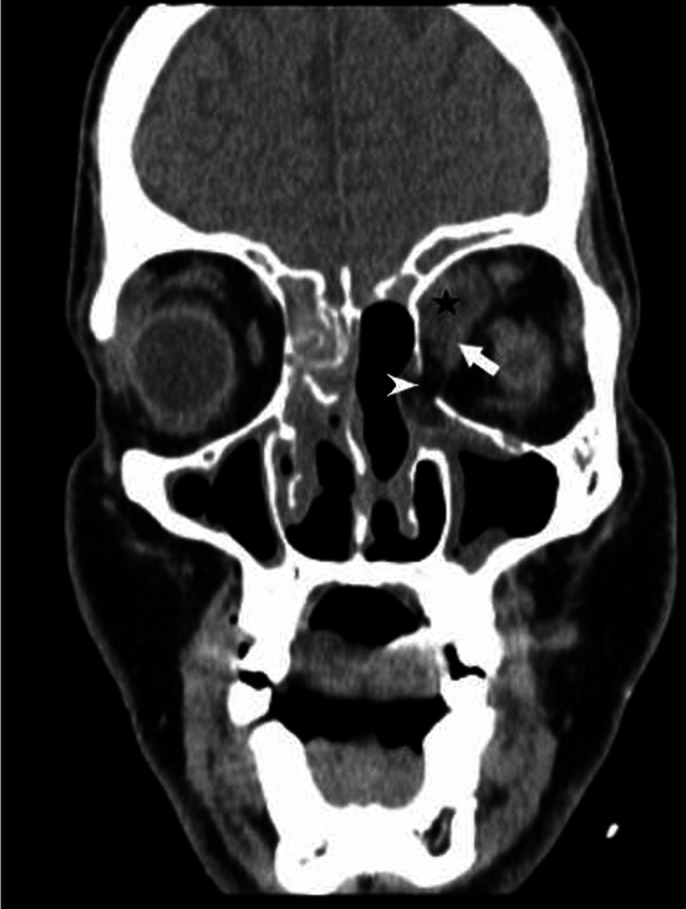
NCCT PNS (coronal section) showing bony dehiscence (arrowhead), intraorbital soft tissue component (star) reaching up to the medial rectus (arrow) with loss of fat planes with it. NCCT, non-contrast CT; PNS, paranasal sinus.

Patient complained of repeat swelling and pain in the left eye (Day 14), following which a repeat CT PNS (Figure 4) was performed. It showed an increase in the intraorbital component of the disease with mucosal thickening and hyperdense contents in the sinuses and nasal cavity. Additionally, bony dehiscence of the inferomedial orbital wall was also noted.

Repeat debridement with nasal suctioning was performed. Enucleation was not indicated as the optic nerve, the eyeball and the intraconal compartment were free of the disease.

## Case 2

A 54-year-old male patient, known case of Type 2 diabetes mellitus and hypertension for the past 5 years, recently tested positive for COVID-19 and was hospitalized for the same. His CT chest revealed a severity score of 12/25. After undergoing treatment for about 11 days, he was discharged on oral steroids 40 mg/day on a gradual tapering dose.

6 days after being discharged, he presented with left-sided hemicranial headache with redness and pain in the left eye for the past 2 days.

His ophthalmology exam revealed left-sided periorbital edema with normal ocular movements and vision. He also complained of left-sided nasal obstruction for 4 days.

**Figure 5.**
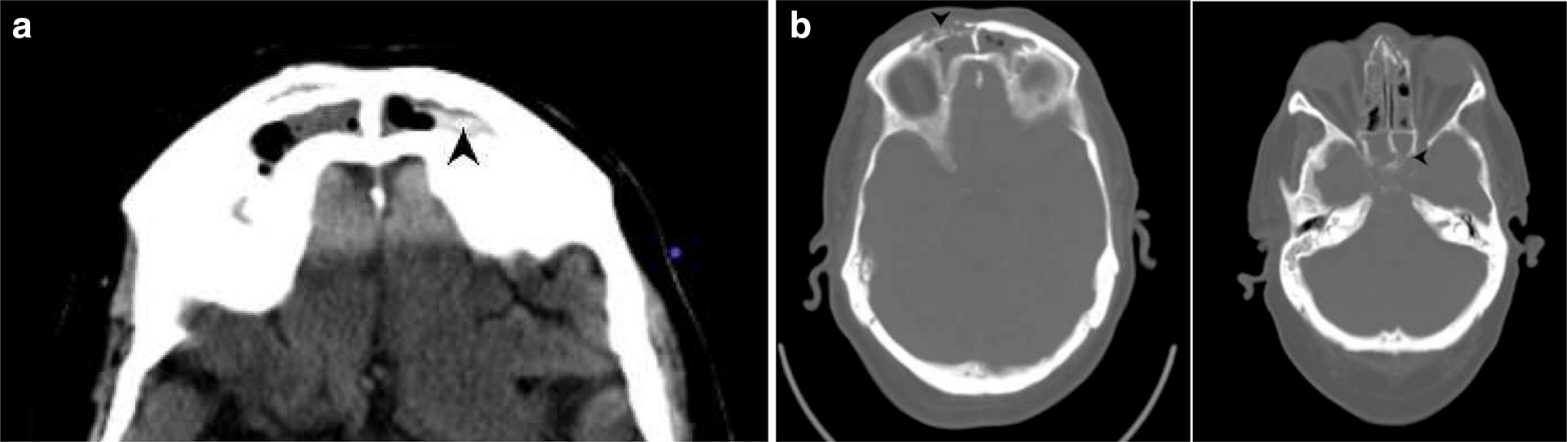
a: NCCT PNS (axial section) - depicting mucosal thickening in bilateral frontal sinuses with hyperdense contents on the left side (arrowhead). b: NCCT PNS (axial section) depicting bony dehiscence of the right frontal and sphenoid sinuses (arrowhead). NCCT, non-contrast CT; PNS, paranasal sinus.

His CT PNS on Day 1 of readmission revealed mucosal thickening with hyperdense contents in bilateral frontal, ethmoid, maxillary, and sphenoid sinuses. Mucosal thickening was also noted in the left nasal cavity. There was bony dehiscence of the walls of the right frontal and bilateral sphenoid sinuses (Figure 5).

**Figure 6.**
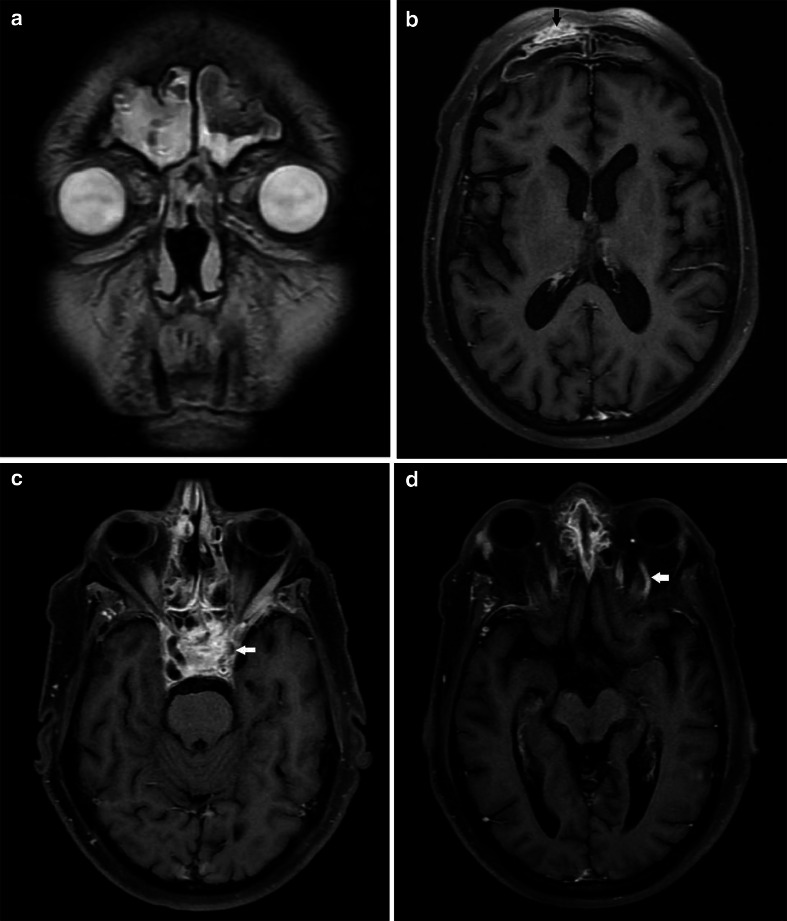
a: *T*_2_ weighted image (coronal section) - hypointense contents within bilateral frontal sinuses - suggestive of fungal sinusitis. b: T1 post-contrast axial section MRI showing abnormal enhancement (arrow) in the bony wall of the right frontal sinus (at the site of bony dehiscence, best depicted on CT, Fig 5b) c: T1 post-contrast axial section MRI - bulky left cavernous sinus with absent flow void in the internal carotid artery (arrow) secondary to cavernous sinus thrombosis. d: T1 post-contrast axial section MRI - enhancing and abnormally enlarged left superior ophthalmic vein (arrow).

Subsequent MRI brain (Figure 6) on Day 2 revealed mucosal thickening in all the sinuses with characteristic T2 hypointense contents within bilateral frontal, ethmoid, sphenoid and left maxillary sinuses, along with enhancement of the sinus walls. There was heterogeneously enhancing soft tissue thickening along bilateral cavernous sinuses (left > right) with non-visualization of flow void of the left cavernous internal carotid artery (ICA). Flow void in right ICA was preserved. The superior ophthalmic vein on the left side appeared dilated. The above features were suggestive of invasive fungal sinusitis with cavernous sinus thrombosis.

Extensive surgical debridement was carried out for the patient and nasal biopsy was sent for microscopic evaluation which revealed mucormycosis.

The patient was initiated on systemic Amphotericin B (5 mg/kg) and antibiotics. Management of cavernous sinus thrombosis by anticoagulants was further carried out under the neurology dept.

## Case 3

A 57-year-old, diabetic, male patient, presented with dysuria and left flank pain for 2 days. He tested positive for SARS-CoV2 RTPCR about 20 days back. His vitals on admission were stable and relevant physical examinations were unremarkable. The patient was currently on tapering doses of oral steroids (initiated on 32 mg/day, and currently on 16 mg/day)

His ultrasound abdomen, performed in another hospital, was normal. As his symptoms did not subside with the initial medications, he was admitted and his RT-PCR for COVID-19 on admission tested negative.

The relevant blood examinations on Day 1 revealed CRP: 96 mg l^−1^, TLC: 13000, UREA: 77 mg/ dl, CREATININE: 2.0 mg dl^−1^, HbA1c: 12.1%

**Figure 7.**
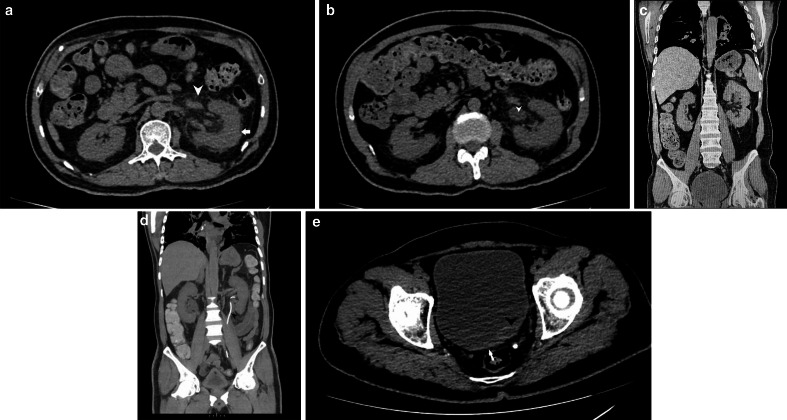
a: NCCT KUB (axial section) depicting an enlarged and bulky left kidney (arrow) with surrounding peri-nephric fat stranding (arrowhead). b: NCCT KUB (axial section) depicting an enlarged left ureter (arrowhead). c: NCCT KUB (coronal section) depicting an enlarged and bulky left kidney (arrowhead) with surrounding perinephric fat stranding. d: NCCT KUB (coronal section, maximum intensity projection image) depicting a left-sided DJ stent (arrowhead). e: NCCT KUB (axial section) depicting bladder wall thickening (arrow) with prominent and hyperdense appearing left VUJ (arrowhead). NCCT KUB, non-contrast CT of Kidneys, ureters, and bladder

He was advised for NCCT KUB (Figure 7) and HRCT chest on Day 2 which revealed a bulky left kidney, with mild-moderate hydronephrosis. There was dense perinephric and periureteric fat stranding with thickened perirenal facias. The left ureter appeared dilated till the vesicoureteric junction. No calculus was noted, however, heterogeneous thickening around the left vesicoureteric junction(VUJ) and the bladder base was noted. Mild hyperdensity was also noted near the left VUJ. A diagnosis of left-sided pyelonephritis with abnormal thickening of the left VUJ was made and the patient was advised for cystoscopic correlation. No contrast study was done due to deranged renal functions.

**Figure 8.**
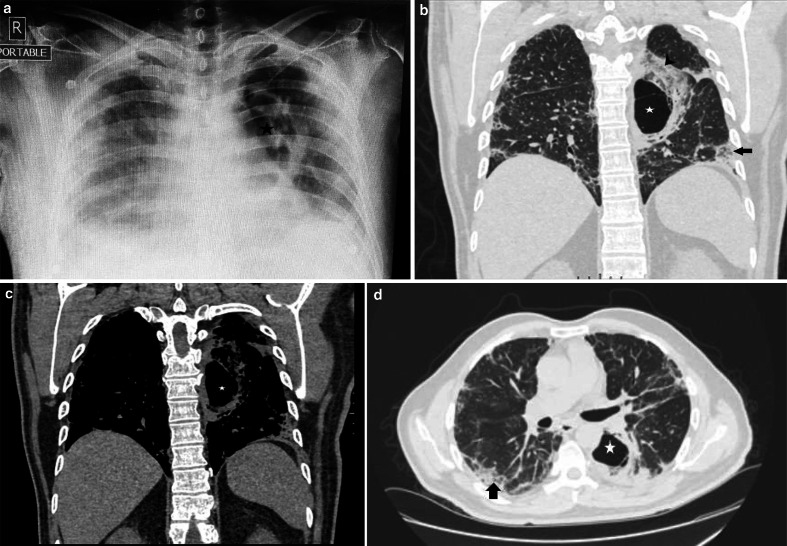
a- X-RAY CHEST (anteroposterior) depicting a thick-walled cavitary lesion in the left lung (star). Inhomogeneous opacities are also noted in bilateral lung fields with a peripheral predominance (COVID pneumonitis) b: HRCT chest (coronal section, lung window) - a large thick-walled cavitary lesion (star) with surrounding ground-glass densities and consolidation (arrowhead) in the superior segment of the left lower lobe. Another lesion with a characteristic reverse halo sign is also noted (arrow). c: HRCT chest (coronal section, mediastinal window)-thick-walled cavitary lesion (star). d: HRCT chest (Axial section, lung window)- depicting resolving changes of covid pneumonitis (arrow) with the above mentioned cavitary lesion (star). HRCT, high resolution CT.

HRCT chest (Figure 8) revealed a large, thick-walled cavitary lesion of size 6 *4 cm in the superior segment of left lower lobe, with few thin septae and surrounding ground-glass opacities and consolidation. Another smaller lesion showing central ground glassing with a peripheral rim of consolidation, giving a characteristic ‘reverse halo sign’, was noted in the posterior basal segment of the left lower lobe.

Few other smaller nodules were also noted in the surrounding left lower lobe.

Features of COVID pneumonitis were also noted in the form of fibrotic bands and septae with patchy subpleural ground-glass opacities and interstitial thickening, seen in both lungs.

**Figure 9.**
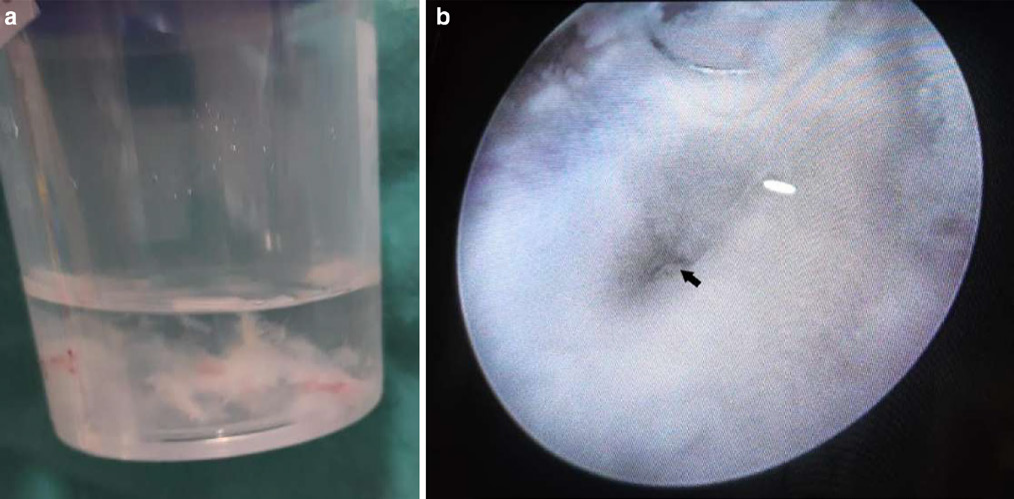
a: Urinary bladder wash depicting fungal hyphae. b: Cystoscopic visualization of the left vesicoureteric junction (arrow), showing blackish discoloration.

The patient was taken for cystoscopy on Day 4, which revealed a fluffy cotton-like ball in the bladder (Figure 9a). Moreover, the left vesicoureteric junction showed blackish discoloration (Figure 9b). Bladder wash fluid was sent for both bacterial and fungal culture, and fluffy balls were sent for histopathological examination (HPE).

Microscopic evaluation of the bladder wash revealed broad, sparsely septate fungal hyphae. And culture revealed Mucorales species.

The patient was started on injection liposomal amphotericin B, 200 mg, i.v. OD for 3 weeks. Additionally, left-sided DJ stenting was also done for him, followed by an ipsilateral nephrostomy through which timely irrigation was performed.

His urine culture was repeated after 3 weeks and following two consecutive negative urine cultures (1 week apart), the patient was discharged on 150 mg, i.v. liposomal amphotericin B every alternate day.

His nephrostomy tube and DJ stent were removed. The patient also undergoes dialysis twice weekly.

## Discussion

With over 161 million cases of the Novel coronavirus (SARS-CoV-2) and over 3 million deaths worldwide, as of May 2021,^1^ the COVID-19 infection has impacted human lives gravely.

Since the start of the SARS CoV-2 pandemic in March 2020, we have been noting either varied manifestations of the disease itself or the numerous complications related to it.

MCR is one such complication, which is being increasingly evident in the Indian subcontinent in the last few months and an active search of the literature revealed no reported case of renal MCR in a post-COVID patient to our knowledge. However, it is clear that several factors are responsible for the sudden increase in incidence of this dreaded infection.

MCR (also called zygomycosis) is a rare invasive fungal infection that mainly infects immunocompromised individuals. After inhalation of spores into the sinuses, the angioinvasive infection gets established via the spores germinating into multiple hyphae in the immunocompromised patient. The pterygopalatine fossa is believed to be the largest reservoir.^2^

The prevalence of MCR in India is approximately 0.14 cases per 1000 population, about 80 times the prevalence in developed countries.^3^

The main risk factors for MCR generally include uncontrolled diabetes mellitus (hyperglycemia stimulates fungal proliferation and also causes a decrease in chemotaxis and phagocytic efficiency which permits the otherwise innocuous organisms to thrive),^4^ hematologic malignancy (acute leukemia in particular), stem cell transplant, solid organ transplant, neutropenia, deferoxamine, and corticosteroid use.^5^ Voriconazole prophylaxis is also an independent risk factor for MCR.^6^

Some risk factors are site-specific, *e.g.* solid organ transplant is associated with a lung infection, uncontrolled diabetes with rhinocerebral mucormycosis.^7^

Recently, an increasing number of cases of this dreaded fungal infection are being seen with COVID-19 patients. But the relationship between the two is largely unclear. A study titled, ‘Mucormycosis in patients with COVID-19’ included 101 cases, 80 percent of whom had pre-existing diabetes mellitus, and 76 percent of whom had received glucocorticoids for the treatment of COVID-19.^8^ This is in keeping with our study, where all patients were diabetic and were undergoing treatment with corticosteroids.

An interesting risk factor for MCR is also the presence of high concentrations of iron in serum.^9^ And it is clear through many studies that iron metabolism plays a central role in regulating MCR infection.^10^

Intracellular iron is bound to ferritin, which is an iron storage protein and is regulated by both, iron availability and inflammation.^11^ An increase in ferritin levels leads to excess intracellular iron that generates reactive oxygen species resulting in tissue damage. This resultant tissue damage leads to the release of free iron into circulation. Iron overload and excess free iron are thus one of the key and unique risk factors for MCR.^12^ As severe COVID-19 is a hyperferritinemic syndrome, we can assume hyperferritinemia (> 500 µg l^−1^) as being one of the predisposing factors for MCR in COVID −19 positive patients.^8^ So, close monitoring of serum ferritin levels is indicated in high-risk patients.

Another risk factor which could be responsible for the recent surge of MCR in COVID-19 patients can be the use of oxygen humidifiers. These were extensively used in the second wave of the disease in the country and can play a role in the transmission of potential nosocomial pathogens via the generation of aerosol particles, which reach deep into the lung immediately after inhalation.^13^

MCR manifests most commonly in the sinuses (39%), lungs (24%), skin (19%), brain (9%), and gastrointestinal tract (7%), and others (6%). Any of these primary forms can lead to disseminated disease (6%) because of its angioinvasive nature.^14^

In Rhino-Orbital-Cerebral mucormycosis (ROCM), clinical manifestations may start with necrosis of the nasal turbinates, palate, or sinuses, which may rapidly progress towards the orbit before reaching intra cranial structures.^15,16^ The most frequent symptoms include headache, fever, amaurosis, proptosis, epistaxis, facial paralysis, and signs of invasion of the trigeminal nerve. Thrombosis of the cavernous sinuses and cranial invasion may be consequences of unresolved rhino-sinus MCR.

The rate of mortality of ROCM is still very high and ranges from 30 to 69%^17^ with isolated involvement of PNS and >80% when brain invasion has occurred. Indicators of poor prognosis include a delay in treatment of more than 6 days, evidence of intracranial invasion, bilateral involvement, invasion of the palate, and the presence of haematological malignancies.^18^

Identification of the aforesaid changes on CT and MRI can help in making an early diagnosis of ROCM. In the early stages, CT shows mucosal thickening in the involved sinuses, with hyperdense contents. Consequently, destruction of medial orbital wall and invasion of rectus muscles, orbital apex, and ipsilateral cavernous sinus may be seen.^19^

MRI provides better visualization of the extent of the disease because of excellent soft-tissue contrast. It depicts the involvement of the orbital soft tissue, infratemporal fossa, intracranial structures, and perineural invasion and vascular obstruction better than CT. The MRI findings^20^ in invasive ROCM include - isointense lesions relative to brain on *T*_1_WI, iso to hyperintense on *T*_2_WI, the fungal element tend to have a low intrinsic signal on *T*_2_WI. On post-contrast images, the devitalized mucosa appears as a non-enhancing tissue giving the appearance of a ‘black turbinate sign’.^21^ Orbital involvement can be observed as an orbital mass and/or thickening of the recti and optic nerve.

Cavernous sinus thrombosis usually results from the spread of infection from the orbit and appears as a filling defect within the enhancing sinus or as a lateral convexity. Other features like narrowing of the carotid artery, carotid arterial wall enhancement, and other intraparenchymal abnormalities like cerebral infarcts, empyema, and meningitis may also be seen.^22^

Cerebral angiography may further reveal vascular occlusion, aneurysmal dilatation, or filling defect.^23^

Pulmonary mucormycosis (PM) most commonly presents as lobar/segmental consolidation. CT initially might simply show a ground glass nodule prior to the development of more extensive lesions. Ground-glass lesions usually progress to consolidation, nodules, or masses.^24^ Occasionally, a cavity can appear at imaging as the initial manifestation. Other vascular findings may include pseudoaneurysm formation and abrupt termination of a pulmonary artery branch.^22^

While most fungal pneumonia shows nonspecific signs at imaging, the ‘reverse halo sign’ has been shown to be a specific sign of mucormycosis, occurring in 19–94% of patients with PM.^22,25^ It is defined as ground-glass opacity surrounded by a rim of consolidation. The sign can also help distinguish between other fungal pneumonias, particularly invasive pulmonary aspergillosis (IPA). Other features that help in differentiating between the two are the presence of multiple surrounding nodules and pleural effusion - both of which are more indicative of PM.^26^

The pulmonary infection can also spread to the pleura, mediastinum, heart, or diaphragm.^27^

In the disseminated form which accounts for 6–9% of total cases; the organ most commonly involved is the lung, with involvement of the kidneys being reported in up to 20% of cases.^28^

Genitourinary MCR in itself is a rare case and its occurrence in a post-COVID patient is by far atypical, with no known case report presented to date to our knowledge.

Renal MCR has been postulated to result from either hematogenous dissemination or via retrograde spread from lower urinary tract infection.^29^

Sonography is usually the initial modality of choice and can depict an enlarged kidney with loss of normal renal echotexture. It can depict hyperechogenic contents (fungal elements) in the urinary bladder and thickened bladder wall.

On CT the affected kidney is bulky, with significant perinephric stranding. Perinephric collections are also common. Hypodense/non-enhancing areas correspond to abscess formation or infarction. Advanced infections may lead to renal infarction and necrosis that appear hypoenhancing on CT and contrast-enhanced MR images.^30^

Fungus balls that occupy the renal collecting system appear as soft-tissue attenuating masses within the collecting system. At MR imaging, they are usually isointense on *T*_1_WI and hyperintense on *T*_2_WI.^27^

In case of involvement of the bladder/vesicoureteric junction (as in our case), upstream hydronephrosis may also be seen.

**Figure 10.**
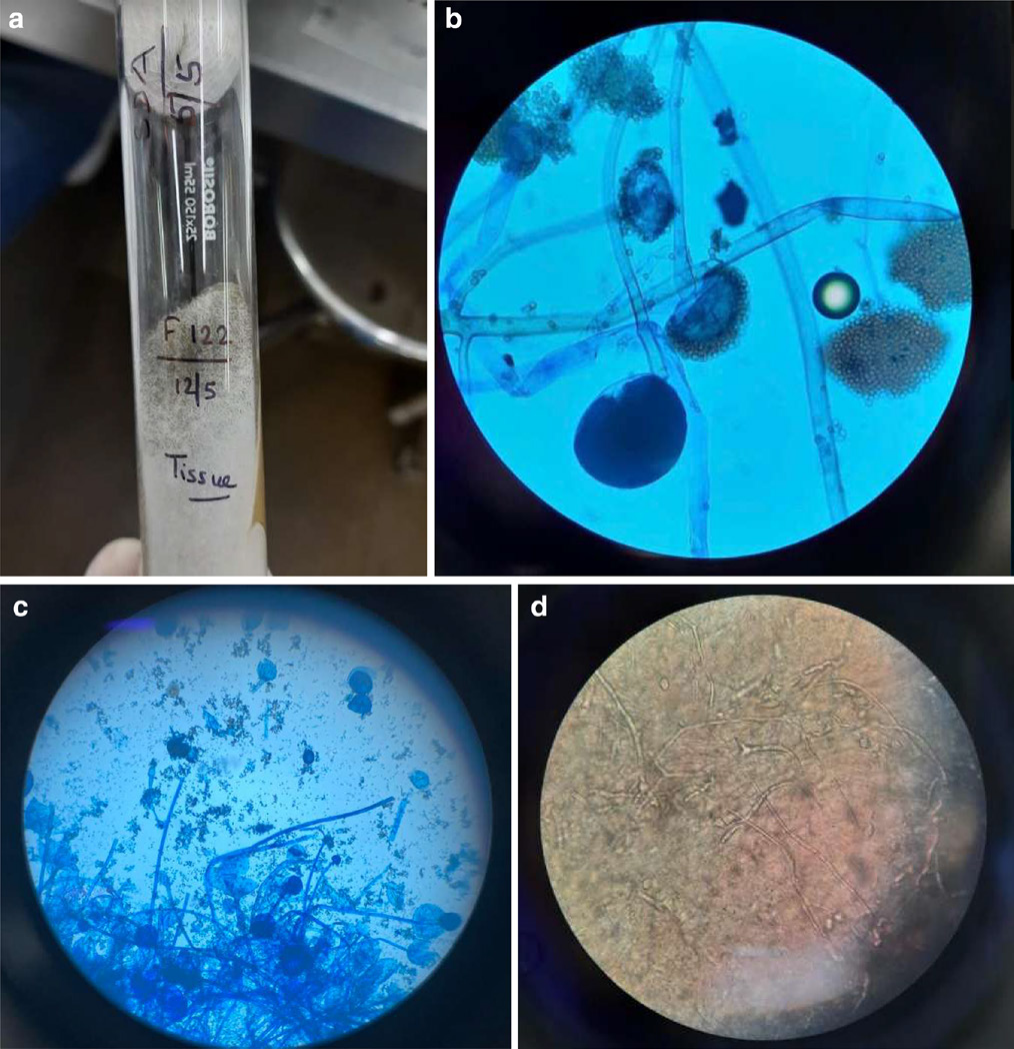
a–d (Microbiology images of mucor, Case 1) - (a)Culture on SDA showing cottony (white to gray) growth. (b, c) lactophenol cotton blue tease mount from SDA showing broad, aseptate hyphae, with an extension of columella into sporangium and aggregation of sporangiospores. (d) tissue sample showing necrotic and edematous tissue with hyphae. SDA, Sabouraud dextrose agar.

The definitive diagnosis of any form of MCR requires a tissue specimen containing the fungal elements. The classic histologic specimen shows large, broad, non-septate, hyphae with right-angled branching and distinct angioinvasion^9^ (Figure 10).

A delay in diagnosis means a worse prognosis for the disease therefore an ‘Early diagnosis’ of MCR holds high importance as it may reduce the need for or extent of surgical resection, disfigurement, and suffering and thereby improve the overall outcome and survival of the patient.

This can be achieved by first, the recognition of host factors (*i.e.* the assessment of a patient’s risk for invasive MCR)- most important being diabetic ketoacidosis, prolonged glucocorticosteroid therapy, immunocompromised status, and a hyperferritinemic state - for this, simple laboratory tests like HbA1c and serum ferritin levels can be performed. Also, concomitant illnesses like TB and HIV should be looked for, as these are quite common in India and can contribute to the immunocompromised state of the patient.

Second, by careful assessment of clinical history and examination (periorbital swelling, diplopia, necrotic eschar in maxillary, facial, or sino-orbital tissues) thirdly, early use of CT and MRI modalities (PNS and brain) and finally adept evaluation of microbiological, histological and cytological preparations should be performed.^31^

The complete management of MCR thus requires a rapid diagnosis, correction of predisposing factors, early treatment with liposomal Amphotericin B, and surgical debridement. A delay in diagnosis means a worse prognosis for the disease.

Also, hyperglycemia should be strictly controlled and the use of glucocorticoids in mild cases (without hypoxemia) or the utilization of higher doses of glucocorticoids should be avoided. Also, in the absence of a clear benefit, drugs targeting immune pathways such as tocilizumab should be discouraged.^32^

## Conclusion

Although all the sequelae and complications of COVID-19 are yet to be documented and described, an increase in secondary infections is being increasingly recognized worldwide. Patients with COVID-19 are more vulnerable to fungal infection such as MCR because of the compromised immune system with decreased CD4+ and CD8+ lymphocytes, associated comorbidities such as diabetes mellitus, and the use of immunosuppressive therapy for the management in moderate to severe cases.^33^ In a review, Song et al noted that fungal infections are more likely to develop during the middle and later stages of COVID-19 infection.^34^

MCR being angioinvasive invades blood vessels upon inhalation and germination of spores and leads to subsequent tissue infarction, necrosis, and thrombosis. This fungal infection is life-threatening and can lead to disseminated disease in no time. Therefore, physicians should follow a multidisciplinary approach focussing on prevention (with strict glycemic control and prudent use of steroids and immunosuppressive agents), identification of high-risk patients (raised serum ferritin levels, immunocompromised status, post-transplant patients, etc.) prompt diagnosis (with laboratory and radiological investigations), treatment with antifungals, and appropriate surgical consultation and treatment.

Radiology plays an important role in diagnosis, estimating the extent of the disease, and assessing the response of treatment. Under strong clinical suspicion, MRI brain and PNS should be done to reach a diagnosis for ROCM. CT cuts could be taken to rule out bony discontinuity and destruction. Thickened mucosal lining and sinus opacification are universally present. Serial radiological investigations are required to assess progression and extent.^35^ HRCT chest and urography should be performed in cases of pulmonary and genitourinary mucor mycosis respectively.

## Learning objectives

1. MCR is a deadly disease and should be aggressively searched among patients with hyperglycemia either due to heavy use of corticosteroids or due to pre-existing diabetes mellitus type II.
2. The use of corticosteroids and immune suppressants could increase the risk of fungal infections, and therefore should be used judiciously.
3. Whenever there is clinical suspicion for MCR, radiological evaluation with CT and MRI (especially of paranasal sinuses and brain) can help reach a diagnosis sooner. It can also aid in better surgical planning by depicting accurate anatomic involvement of the disease.
4. Early diagnosis and treatment, which involves extensive surgical debridement and liposomal Amphotericin B are necessary to reduce overall mortality in the patient.
5. Hyperglycemia and hyperferritinemia are both important risk factors for MCR. Their timely monitoring can help in identifying ‘at risk’ patients sooner, aiding in better management.
6. Also, we need to propagate the use of clean masks, sterile water for oxygen humidifiers and strengthen the overall ‘infection prevention and control policy’ of the health-care facilities.
